# Surface Characterization of Asymmetric Bi-Soft Segment Poly(ester urethane urea) Membranes for Blood-Oxygenation Medical Devices

**DOI:** 10.1155/2012/376321

**Published:** 2011-11-13

**Authors:** Mónica Faria, Vítor Geraldes, Maria Norberta de Pinho

**Affiliations:** ICEMS and Department of Chemical and Biological Engineering, Instituto Superior Tecnico, Technical University of Lisbon, 1049-001 Lisboa, Portugal

## Abstract

Asymmetric bi-soft segment poly(ester urethane urea) (PEUU) membranes containing polycaprolactone (PCL) as a second soft segment are synthesized with PCL-diol ranging from 0% to 15% (w/w). Bulk and surface characteristics of the PEUU membranes were investigated by scanning electron microscopy (SEM), static water contact angles, and surface streaming potentials and were correlated to hemocompatibility properties, namely, hemolysis and thrombosis degrees. SEM analysis reveals PEUU membranes with asymmetric cross-sections and top dense surfaces with distinct morphologies. The increase in PCL-diol content yields PEUU membranes with blood-contacting surfaces that are smoother, more hydrophilic, and with higher maximum zeta potentials. The results obtained in this work give no evidence of a correlation between hydrophilicity/zeta potentials and the hemolysis/thrombosis degree of blood-contacting surfaces of the PEUU membranes. In contrast, other hemocompatibility aspects reveal that the more hydrophilic membranes are associated with lower platelet deposition and inhibition of extreme states of platelet activation.

## 1. Introduction

Polyurethanes (PUR) are a class of polymers with widespread use in medical devices because of their reasonable bio- and hemocompatible properties, in association with a good flex life, mechanical strength, tear resistance, and versatility in tailoring the final bulk and surface properties [1]. At the nanometer scale PURs are not homogeneous materials: a microphase segregation process leads to the formation of a two-microphase structure with regions enriched in either polyurethane/urea hard or polyether, polyester or polybutadiene soft, amorphous segments. This segregation is thermodynamically driven by unfavorable interactions between polar urethane/urea and relatively nonpolar macroglycol segments. The heterogeneous morphology of polyurethanes is perturbed at an interphase where the chemical composition and morphology ultimately attained is a balance between bulk and interfacial interactions. In bi-soft segment PURs, a second soft segment is used to extend an isocyanate terminated prepolymer that contains the first soft segment. Apart from the possibility of occurrence of different extents of phase separation between soft and hard segments, the two soft segments can also show different extents of phase separation, opening up new possibilities for tuning bulk and surface membrane properties [2–9].

The PUR composition implies a wide diversity of surface characteristics, which in turn are of prime importance when dealing with an eventual use of PURs as blood-contacting materials. The PUR surface layers that are in contact with the blood may differ compositionally from the bulk as polymer chains are mobile and can rearrange in response to interfacial forces [10]. The interaction between a foreign material and blood starts at the material's surface, giving rise to a sequence of events which include protein adsorption, adhesion/activation of platelets, adhesion of leucocytes, blood coagulation, and complement activation; therefore, it is increasingly recognized that the biomedical performance of polymers in blood-contacting devices is a function not only of the bulk properties, but particularly of the nature and the topography of the surface. In this framework of correlating the structural versatility of bi-soft segment polyurethanes and their surface interaction with blood, there are intense developments in relation to dense films or membranes with symmetric cross-sections [2, 3, 11–16]. In contrast, for bi-soft segment polyurethane membranes with asymmetric cross-sections there is very scarce literature, despite their very different surface properties [17].

In this work, asymmetric bi-soft segment poly(ester urethane urea) (PEUU) membranes were prepared and characterized with view to an application in extracorporeal blood-oxygenation medical devices. For the synthesis of the PEUU membranes we use a triisocyanate terminated prepolymer (PUR) containing poly(propylene oxide) (PPO) as the first soft segment which has been revealing good membrane-forming characteristics in the development of membranes with reasonable hemocompatibility [11, 16]. The second soft segment of poly(*ε*-caprolactone) diol (PCL diol) was selected as it contributed to the enhancement of hemocompatibility in regard to minimal platelet deposition and inhibition of extreme stages of platelet activation [18]. The PEUU membranes blood-contacting active layers are characterized through scanning electron microscopy (SEM), water contact angles and surface streaming potentials in order to correlate their surface properties with hemocompatibility performance. 

## 2. Materials and Methods

### 2.1. Materials

Segmented PEUUs were synthesized in bulk by extending a poly(propylene oxide)-(PPO-) based polyurethane prepolymer (PUR) with three isocyanate terminal groups (Companhia Petroquímica do Barreiro, Barreiro, Portugal; MW: 3500; 3.6 wt% of isocyanate groups) with a second prepolymer-poly(*ε*-caprolactone) diol (PCL diol) (Aldrich; molecular weight 530; 6.4 wt% of hydroxyl groups). Dimethyl formamide (DMF) (p.a. grade, Aldrich) and diethyl ether (DEE) (p.a. grade, Aldrich) were used as solvents and stannous octoate (Aldrich) as a catalyst.

### 2.2. Membrane Preparation

Four integrally skinned asymmetric membranes, PEUU 100, PEUU 95, PEUU 90 and PEUU 85, were prepared by a modified version of the phase inversion technique. In this process, the PPO-based prepolymer, PUR, with three isocyanate terminal groups and the PCL diol with two terminal hydroxyl are dissolved in a homogeneous phase solvent system of DMF and DEE at a weight ratio DMF/DEE of 3. The polymerization reaction is carried out in an inert (nitrogen) atmosphere at room temperature for 2 h with continuous stirring and was catalyzed by stannous octoate (2 drops). Four casting solutions with PUR/PCL diol weight percentage (wt.%) ratios of 100/0, 95/5, 90/10, and 85/15 and equivalent NCO : OH (wt%) ratios of 100 : 0, 91 : 9, 83 : 17, and 76 : 24 were prepared in a solvent mixture of DMF/DEE, where the weight ratio of total polymer to total solvent was kept constant and equal to 2/3. The solutions were cast onto a glass plate at room temperature with a 250 *μ*m casting knife and after 30 s of solvent evaporation time were quenched into a gelation bath of deionized water at 23 ± 2°C to yield membranes PEUU 100, PEUU 95, PEUU 90, and PEUU 85. After 12 h in the gelation bath the resulting membranes were detached from the glass plate, thoroughly washed with deionized water (conductivity below 0.2 mS/cm) to remove any solvent traces, and finally dried in an oven at 40 ± 3°C for 36 h. Before casting the PEUU membranes, ATR-FTIR spectra were taken for all the solutions to make sure that none showed a peak centered at approximately 2260 cm^−1^ corresponding to the asymmetric isocyanate stretching mode. This was an indication that all isocyanate groups have reacted with the hydroxyl groups of PCL diol, and/or with water in ambient air and, subsequently, with the resulting amine groups, forming urethane and urea linkages.

**Figure 1:**
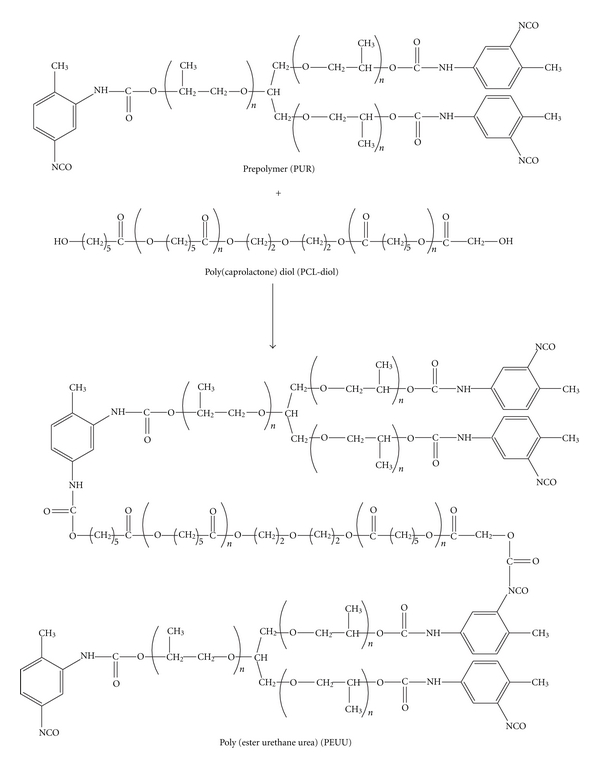
Preparation of segmented integrally skinned asymmetric PEUU membranes.

The PEUU 100 membrane is prepared in the absence of PCL diol and therefore contains only one type of soft segment (PPO); the other three membranes, PEUU 95, PEUU 90, and PEUU 85, contain two soft segments: PPO and PCL.  Figure 1 shows the reaction scheme for the synthesis of bi-soft segmented integrally skinned asymmetric PEUU membranes.

### 2.3. Scanning Electron Microscopy (SEM)

Scanning electron microscopy (SEM) was carried out with a JSM-7001F (JEOL, Japan). The bottom porous, top dense blood-contacting surfaces and cross-sections of the PEUU membranes were gold coated before the imaging, and the images of the membrane surfaces with scanned areas of 32 *μ*m × 24 *μ*m and magnification of 3500 were obtained at a scanning voltage of 10 kV. Quantitative analysis of the surface area occupied by the polymer matrix as well as the range of diameters of the pores was calculated using the software ImageJ version 1.4.3.67 (NIH Image, USA) [19].

### 2.4. Blood-Contacting Surface Hydrophilicity—Static Water Contact Angles

The water contact angle measurements were carried out by the pendant drop method using a video camera (jAi CV-A50) mounted on a Wild M3Z microscope to record the drop image. The video signal was transmitted to a frame grabber (Data Translation model DT3155), with the image acquisition and analysis performed on a computer running the ADSA-P software (Axisymmetric Drop Shape Analysis, Applied Surface Thermodynamics Research Associates, Toronto, Canada). Mean static contact angle values were obtained with distilled water by 10 measurements on different positions of the top dense blood-contacting surface of randomly chosen samples of the PEUU 100, PEUU 95, PEUU90, and PEUU 85 membranes.

### 2.5. Blood-Contacting Surface Charge—Surface Streaming Potential

The streaming potential on the blood-contacting surface of the PEUU membranes was measured by an Electrokinetic Analyzer (Electrokinetic Analyzer EKA, A. Paar GmbH). Two samples of each membrane were placed on the membrane holder with their active layers or blood-contacting surfaces facing each other separated by a spacer at a distance of approximately 1 mm. Using the Clamping Cell a pressure ramp from 0 to 600 mbar was employed to force the electrolyte solution through the cell. The pH dependence of the zeta potential for the blood-contacting surfaces of the PEUU membranes was determined from streaming potential measurements in an electrolyte solution of 1 × 10^−3 ^mol/L KCl with pH adjusting solutions 0.1 M HCl and 0.1 M NaOH. The three aqueous solutions were prepared from deionized water, and the streaming potentials were converted to zeta potentials using the Helmholtz-Smoluchowski equation [20]. Each value of the zeta potential at a given pH value represents an average value over at least five individual measurements. The evaluation and interpretation of the zeta potential versus pH plots is based on the following general findings and concepts: polymers without dissociating surface functions can be highly charged in diluted KCl solutions due to preferential adsorption of OH^−^ ions. The isoelectric point (IEP) for these polymer surfaces obtained when decreasing the solution pH by addition of HCl is found at pH 4.0 ± 0.1 [21, 22]. It is independent of any properties of the polymer beyond the absence of Bronsted functions and almost independent of the KCl solution concentration. The occurrence of the IEP in the acidic pH range is attributed to the preferential OH^−^ adsorption; that is, it reflects the ratio of the OH^−^/H^+^ adsorption [23–25]. Another conclusion can be drawn from the extreme zeta potential value obtained for its dependence on the solution concentration of surface charge-determining ions if the polymer surface is smooth and nonporous and does not contain dissociating surface functions Since the adsorption of the charge-determining ions onto indifferent surface sites occurs in competition with the adsorption of water, the maximum zeta potential correlates with the hydrophobicity of the surface [26].

### 2.6. Hemocompatibility Evaluation

The hemocompatibility evaluation was carried out *in vitro* according to the ISO 10993-4:2002 standard [27]. All tests used pooled rabbit blood anticoagulated with acid citrate dextrose (ACD) solution, at a blood/ACD ratio of 9 : 1. Blood was collected by standard venipuncture (18G needles) from normal, healthy rabbits.

#### 2.6.1. Hemolysis

Hemolysis was assessed according to the ISO 10993-4:2002 standard [27] following a method detailed by us before [11] which is based on the ASTM F 756-00 standard recommended in the ISO 10993-4 : 2002 standard [28]. Briefly, triplicate samples of each membrane were studied before and after extraction with phosphate-buffered saline (PBS, 0.01 M, pH 7.4) for 48 h at 37°C, under static conditions (titled unextracted membranes and PBS-extracted membranes, resp.). After 4 h of contact with static blood at 37°C, the hemoglobin (Hb) released was quantified (cyanmethemoglobin method) [29]. From the Hb concentration released, the hemolysis index was calculated and expressed as a percentage in relation to the Hb concentration in the positive control (blood plus water), after subtracting a blank (blood plus PBS) from each Hb concentration. The membranes are classified according to the hemolysis index (HI) as nonhemolytic (HI 0–2%), slightly hemolytic (HI 2–5%), or hemolytic (HI >5%) [28].

#### 2.6.2. Thrombosis

Thrombosis was evaluated through an assay described by us in previous studies [11] which is based on a version of the method proposed by Imai and Imai and Nose [30] and Allmer et al. [31]. In brief, the thrombus mass formed on the top dense blood-contacting surface of the PBS-extracted membranes was determined gravimetrically after different contact times (one membrane sample, in triplicate, for each contact time) with recalcified, static blood. For each contact time of 15 mins, 25 mins, 35 mins, 45 mins, and 55 mins, a filter paper disk (in triplicate), which followed this procedure in parallel but in the absence of a membrane sample and of blood, was used as a blank. From the thrombus mass formed on each sample, a thrombosis degree was calculated as a percentage of the thrombus mass formed on the positive control (glass, evaluated in triplicate for each contact time) after subtracting the blank from each thrombus mass. A thrombosis degree of 100% was assigned to the positive control (glass).

## 3. Results and Discussion

Four different PEUU membranes prepared with PUR/PCL-diol in wt% ratios of 100/0, 95/5, 90/10, and 85/15 were designated by PEUU 100, PEUU 95, PEUU 90, and PEUU 85. They were white in color, very flexible, and with thicknesses ranging from 40–65 *μ*m (measured and averaged on 6 sampling points for each membrane).  Table 1 shows the chemical compositions of the PEUU membranes in terms of PUR/PCL-diol (wt.%) and the water static contact angles measured on the top dense blood-contacting surface of the PEUU membranes.

**Figure 2:**
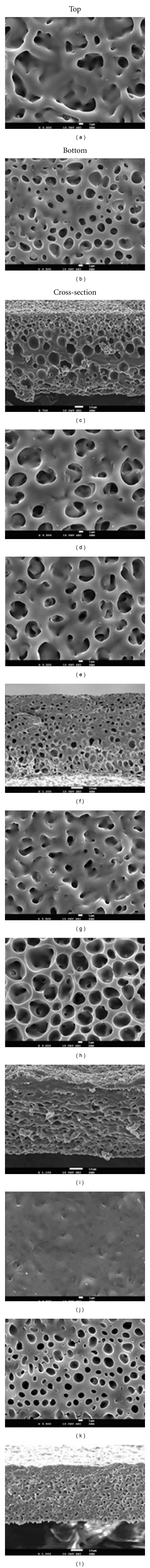
SEM images of PEUU 100 (a, b, and c), where, (a) active layer, (b) bottom surface, (c) cross-section, PEUU 95 (d, e, and f) where, (d) active layer, (e) bottom surface, (f) cross-section, PEUU 90 (g, h, and i) where, (g) active layer, (h) bottom surface, (i) cross-section, PEUU 85 (j, k, and l) where, (j) active layer, (k) bottom surface, (l) cross-section of the membranes.

Figure 2 shows the SEM images (scan area 32 *μ*m × 24 *μ*m) of the surfaces of the top dense and bottom porous layers and of the cross-section of PEUU 100, PEUU 95, PEUU 90, and PEUU 85 membranes casted from solutions with varying concentrations of PUR and PCL-diol. The left and center columns of Figure 2 refer to the top dense blood-contacting surfaces and the bottom porous gas contacting surfaces of the PEUU membranes, respectively.  Figure 2(a) shows the SEM image of the dense layer of the PEUU 100 membrane as a very nonuniform surface.  Figure 2(b) shows the bottom porous layer of the same membrane (PEUU 100) where depressions with diameters ranging from 1.0 *μ*m to 3.5 *μ*m are dispersed in a nonuniform manner throughout the polymer matrix that covers approximately 70% of the total surface area. In Figure 2(d), the SEM image of the dense layer of the PEUU 95 membrane shows round/oval depression areas with diameters ranging from approximately 1.5 to 5.5 *μ*m and dispersed in a polymer matrix with approximately 50% of the total surface area. The porous surface of this PEUU 95 membrane (Figure 2(e)) shows depression areas with roughly the same shape and sizes ranging from 3.0 *μ*m to 5.5 *μ*m in diameter and dispersed throughout the polymer matrix in a similar manner as the top dense surface. In Figure 2(g), the top dense layer of the PEUU 90 membrane shows very small depressions with an average diameter of 1.5 *μ*m and dispersed in a polymer matrix occupying approximately 66% of the total surface area.  Figure 2(h) shows the bottom porous surface of membrane PEUU 90. There are large and very irregular depressions with diameters ranging from 3.5 *μ*m to 5.5 *μ*m that lead to a very small polymer matrix surface area of approximately 35% of the total surface area. There is a very big difference in the surface morphology of the top dense and bottom porous layer of this membrane containing 90 wt% of PCL-diol. In Figure 2(j), the SEM image of the dense layer of the PEUU 85 membrane shows a very homogeneous surface.  Figure 2(k) shows the porous surface of the PEUU 85 membrane with pores of round shape with diameters ranging approximately from 0.5 *μ*m to 2.0 *μ*m which are dispersed throughout the polymer matrix and with this polymer matrix covering a surface area of 70%. For the PEUU 100, PEUU 90, and PEUU 85 membranes, the difference in the surface morphology of the top layer and of the porous sub layer is very clear with top layers that are much denser and have higher polymer concentration than the bottom surface. For the membrane with the smallest content of PCL diol, 5 wt.%, the differences in morphology between the two surfaces are less evident. The column on the right of Figure 2: Figures 2(c), 2(f), 2(i), and 2(l) show the cross-sections of the PEUU 100, PEUU 95, PEUU 90, and PEUU 85 membranes, respectively.  Figure 2(c) depicts a structure with large round-like pores in the center which appear to become smaller towards the surfaces of the PEUU 100 membrane. In Figure 2(f), we can see for the PEUU 95 membrane the round-like pores of bigger dimensions near the bottom porous surface which seem to become smaller towards the top active layer. For the PEUU 100 and PEUU 95 membranes the pores seen in the cross-sections have a round-like structure and seem to be somewhat smaller for the PEUU 95 membrane than for the PEUU 100 membrane.  Figure 2(i) shows the cross-section image for the PEUU 90 membrane, and unlike the PEUU 100 and PEUU 95 membranes, the pores do not seem to have a round-like shape and are instead characterized by an anisotropic shape. In the near active layer surface region there seem to be less pores than in the center and the near bottom porous surface of the PEUU 90 membrane.  Figure 2(l) shows the cross-section of the PEUU 85 membrane, and like for the PEUU 90 membrane the pores are of different shapes unlike the round-like pores in the PEUU 100 and PEUU 95 membranes. Although similar in shape to the ones present in the PEUU 90 membrane, the pores in the PEUU 85 membrane seem to be smaller. 

The hydrophilicity of the blood-contacting dense surface of the bi-soft poly(ester urethane urea) membranes was characterized by static water contact angles.  Table 1 shows the contact angles on the top dense blood-contacting surfaces decreasing from approximately 71° to 66° to 63° and finally to 59° with the increasing content of the second soft segment, PCL-diol, from 0 wt.% to 5 wt.% to 10 wt.%, and finally to 15 wt.%, respectively. The smallest contact angle, approximately 59°, indicating the most hydrophilic surface, was measured for the PEUU 85 membrane which contains the highest PCL-diol content, 15 wt.%, while the most hydrophobic blood-contacting surface was verified for the PEUU 100 membrane with no PCL-diol content, and that contains only one type of soft segment—PPO.

**Figure 3:**
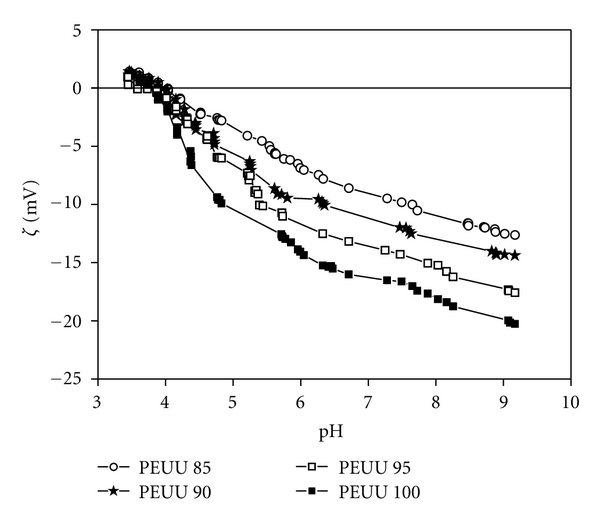
Zeta potential versus pH plot for the blood-contacting surface of the PEUU membranes.

**Figure 4:**
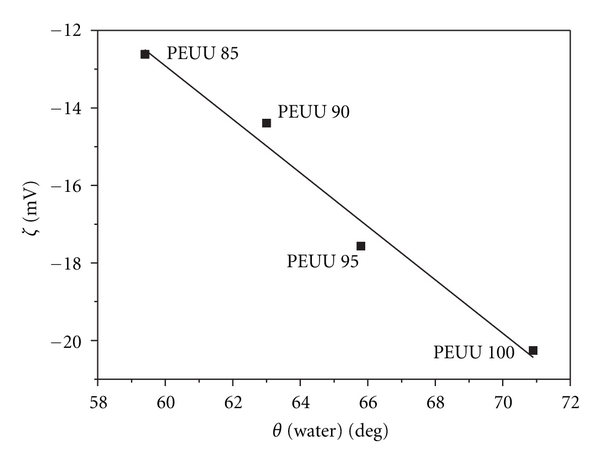
Correlation between water contact angle and maximum zeta potential observed in zeta potential versus pH plots at alkaline pH values (NaOH addition, 10^−3^ M KCl background electrolyte) for the blood-contacting surface of the PEUU membranes.

The pH dependence of the zeta potential was determined from the streaming potential measurements for the four blood-contacting dense surface of the bi-soft poly(ester urethane urea) membranes.  Figure 3 shows the zeta potential versus pH plot for the blood contacting dense surface of the PEUU membranes. The isoelectric point (IEP) for all the PEUU membranes was found at a pH of approximately 3.9 and the maximum zeta potentials observed were approximately of −20 mV, −17 mV, −14 mV, and −12 mV for the PEUU 100, PEUU 95, PEUU 90, and PEUU 85 membranes, respectively. For all PEUU membranes, both the IEP at approximately a pH of 3.9 and the absence of a plateau range in the zeta potential versus pH plot are typical characteristics of polymers bearing no dissociating groups. Due to the hydrophobic character of the poly(ester urethane urea) membranes, preferred absorption of the chloride anions is observed which gives a negative zeta potential in the range of pH = 4–10. Further, a maximum of the zeta potential is observed with increasing solution pH caused by NaOH addition. This extreme value is attributed to the superposition of increasing interfacial charge due to preferential OH^−^ ion adsorption and double-layer compression with increased NaOH solution concentration [32]. In the past, studies have shown for smooth, nonporous polymer membranes, the existence of a linear correlation between the water contact angle and the maximum zeta potential; this is explained by the fact that the charge formation at the interface by ion adsorption occurs in competition with the adsorption of water molecules [22, 33].  Figure 4 shows the correlation between water contact angles (Table 1) and maximum zeta potential observed in zeta potential versus pH plots at alkaline pH values (NaOH addition, 10^−3^ M KCl background electrolyte) for the blood-contacting surface of the PEUU membranes. As expected, a correlation between decreasing water contact angle and decreasing maximum zeta potential was confirmed for the PEUU membranes. The most hydrophilic membrane, PEUU 85, which has the lowest contact angle, approximately 59°, exhibits the highest value of extreme zeta potential, approximately −12 mV, while the most hydrophobic membrane, PEUU 100, for which the highest contact angle was observed, approximately 71°, has the lowest extreme zeta potential value, approximately −20 mV. Such a linear correlation enables us to predict the hydrophobicity/hydrophilicity nature of the PEUU membranes by analyzing the zeta potential values and vice versa. Furthermore, it opens the possibility of estimating the values of extreme zeta potential and of water contact degrees of PEUU membranes containing different PUR/PCL compositions.

**Figure 5:**
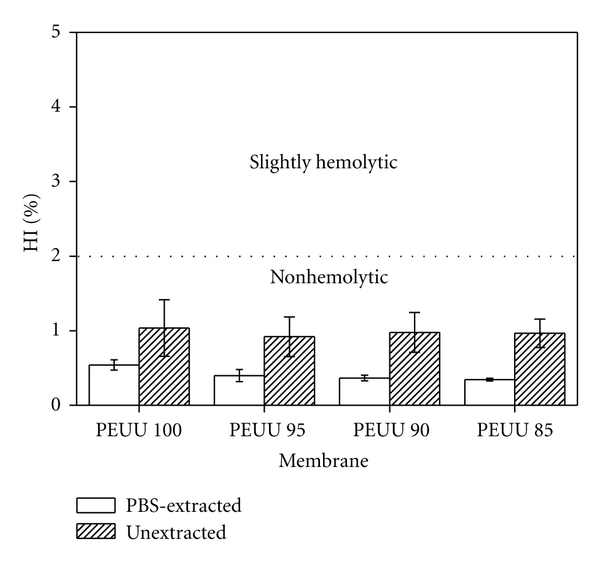
Hemolysis index (HI) of PEUU membranes before and after extraction with PBS. HI between 0 and 2% indicate non hemolytic membranes. The mean hemolysis degrees of the PBS-extracted samples were not significantly different from each other (one-way ANOVA, *P* > 0.05).

Figure 5 shows the results of the hemolysis essay for the PEUU membranes. Damage to the membrane of red cells, as a result of blood exposure to foreign materials, can be evaluated by the hemolysis test. According to ASTM F-756 [28], materials can be labeled nonhemolytic when the Hemolytic Index (HI) is between 0–2, slightly hemolytic when HI is between 2–5, and hemolytic when HI > 5. All of the PEUU membranes were nonhemolytic both before and after PBS extraction for a contact time with blood of 3 h. The differences between the PEUU membranes containing only one type of soft segment (PEUU 100) and the membranes containing two types of soft segments (PEUU 95, PEUU 90, and PEUU 85) were not statistically significant (ANOVA, *P* = 0.05). The hemolysis degree did not vary regularly with PCL content, and the extraction with PBS decreased considerably the hemolysis degree. Since this hemolysis assay was performed with static blood, which excludes the occurrence of hemolysis due to mechanical damage to the erythrocyte's membrane, this difference pointed to the possibility that a fraction of the hemolysis degree detected could be due to foreign substances extracted with PBS (residuals of catalyst, solvent, and oligomers). However, when these extracts were assayed, no hemolysis could be detected, probably due to lack of sensitivity of the method. All subsequent hemocompatibility tests were then carried out with PBS-extracted membranes.

**Figure 6:**
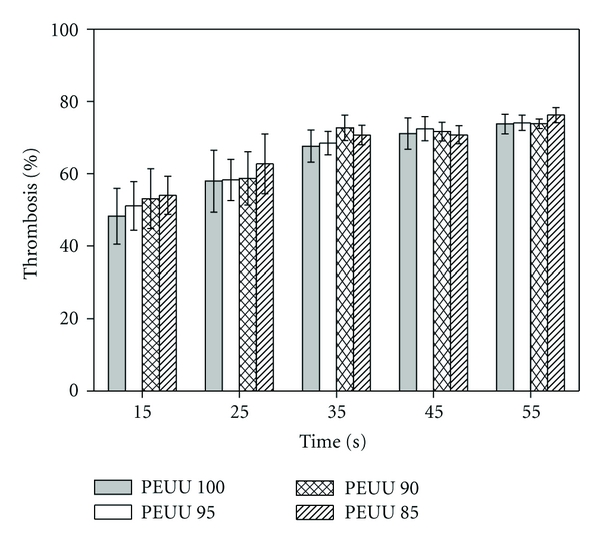
Percentage of thrombosis on the blood-contacting surface of the PBS-extracted PEUU membranes for different contact times with blood (glass: 100%, top line of the graph).

Figure 6 shows the percentage of thrombus formation on the top dense blood-contacting surface of the PEUU membranes for different contact times, admitting that the thrombosis degree on a glass surface is of 100% (positive control). For each and every one of the blood contact times evaluated 15 s, 25 s, 35 s, 45 s, and 55 s, there was no statistical difference in the extent of clot formation on the blood-contacting surface of all PEUU membranes, PEUU 100, PEUU 95, PEUU 90, and PEUU 85. For the shortest contact times with blood (15 min, the earliest time at which it was possible to quantify the thrombus formed), the whole set of membranes showed thrombosis degrees of 48–54%. The thrombosis degree of all the PEUU membranes was highest for a contact time of 55 min (73–76%) but has still not reached that of the positive control (glass surface with thrombosis degree of 100%). For contact times with blood between 45 min and 55 min, the thrombosis degree for all the PEUU membranes was approximately between 71% and 76%, and statistical analysis (one-way ANOVA, *P* > 0.05) did not show significant differences. This may reveal that the highest thrombosis degree is reached after a contact of 45 minutes between blood and the blood-contacting surface of the PEUU membranes, and that at least for the following 10 minutes (blood contacting time of 55 mins) remains approximately constant.

In a previous investigation, Besteiro et al. [16] casted PEUU membranes with the same chemical composition as the ones addressed in this work by the solvent evaporation method of polymeric solutions with PUR/PCL ratios of 100/0, 95/5, 90/10, and 75/25 and a single solvent (toluene). These membranes had symmetric dense cross-sections and the blood-contacting surfaces were characterized by contact angles and by hemocompatibility essays of hemolysis, thrombosis, and platelet adhesion. No firm correlation was obtained between the contact angles and the hemocompatibility properties. In contrast with that, membranes casted as reported in this work, by the phase inversion technique, with the same DMF/DEE solvent system and PUR/PCL ratios, described in Table 1, yielded asymmetric cross-sections and dense top layers (blood-contacting surface) that upon characterization by Atomic Force Microscopy revealed a strong correlation between surface properties and platelet deposition. In fact, the top dense layers became smoother, and their submicron average roughness decreases monotonically by a factor of five to approximately 1 nm as the PCL-diol content increases from 0 wt.% to 15 wt.%. In the same work, the platelet/membrane surface interactions that are quantified by the global parameters of platelet deposition and platelet coverage and are correlated to the submicron average roughness parameter that in turn is controlled through the PCL-diol content of the membrane casting solutions. Furthermore, progressive stages of platelet activation depend on the membrane surface morphologies associated with different PCL contents as extreme states of platelet activation, spread, and fully spread, are inhibited in membranes casted from solutions with 15 wt.% of PCL-diol and the fully spread stage is already inhibited for intermediate PCL-diol contents of 5 wt.% and 10 wt.% [18]. 

## 4. Conclusions

The poly (ester urethane urea) membranes prepared by the modified phase inversion technique under the same casting conditions displayed asymmetric integrally skinned cross-sectional structures that were tailored upon the variation of the PCL-diol content. The characterization of the PEUU membranes through SEM analysis gives evidence that the top dense blood-contacting surfaces become smoother and more dense with increasing concentrations of PCL-diol while static water contact angles and surface streaming potential measurements reveal that the increase in PCL-diol content yields more hydrophilic PEUU membranes with higher maximum zeta potentials. Despite the nonsignificant differences in the hemolysis index and thrombosis percentage between the PEUU membranes containing different PCL content, the more hydrophilic membranes are associated with lower platelet deposition and inhibition of extreme states of platelet activation. Further investigation of local charge distribution and surface energies should be carried out to elucidate the correlation between the streaming potential and the eventual liaison with PCL content.

## Figures and Tables

**Table 1:** Molecular composition and water static contact angles of the PEUU membranes.

Membrane	PUR/PCL diol (wt.%)	Water contact angle (°)
PEUU 100	100/0	70.9 ± 0,6
PEUU 95	95/5	65.8 ± 0,8
PEUU 90	90/10	63.0 ± 0,6
PEUU 85	85/15	59.4 ± 1,0
